# Quantifying the effects of landscape and habitat characteristics on structuring bird assemblages in urban habitat patches

**DOI:** 10.1038/s41598-024-63333-z

**Published:** 2024-06-03

**Authors:** Yun Zhu, Yu Liu, Shang Sheng, Jinfeng Zheng, Su Wu, Zhaoyang Cao, Kai Zhang, Yu Xu

**Affiliations:** 1 Key Laboratory of National Forestry and Grassland Administration On Biodiversity Conservation in Karst Mountainous Areas of Southwestern China, School of Life Sciences, Guizhou Normal University, Guiyang, 550001 China; 2 School of Ecology and Nature Conservation, Beijing Forestry University, Beijing, 100083 China; 3 School of Life Sciences, Southwest University, Chongqing, 400715 China

**Keywords:** Birds, Community composition, Habitat fragmentation, Habitat heterogeneity, Species richness, Biodiversity, Community ecology, Conservation biology, Urban ecology

## Abstract

Understanding the determinants of biodiversity in fragmented habitats is fundamental for informing sustainable landscape development, especially in urban landscapes that substantially fragment natural habitat. However, the relative roles of landscape and habitat characteristics, as emphasized by two competing frameworks (the island biogeography theory and the habitat diversity hypothesis), in structuring species assemblages in fragmented habitats have not been fully explored. This study investigated bird assemblages at 26 habitat patches (ranging in size from 0.3 to 290.4 ha) in an urban landscape, southwest China, among which habitat type composition and woody plant species composition varied significantly. Through 14 bird surveys conducted over six breeding seasons from 2017 to 2022, we recorded 70 breeding bird species (excluding birds recorded only once and fly-overs, such as raptors, swallows and swifts), with an average of 26 ± 10 (SD) species per patch. We found that patch area had significant direct and indirect effects on bird richness, with the indirect effects mediated by habitat richness (i.e., the number of habitat types). Isolation (measured as the distance to the nearest patch), perimeter to area ratio (PAR), and woody plant richness did not significantly predict variation in bird richness. Furthermore, none of these factors significantly sorted bird species based on their functional traits. However, the overall makeup of bird assemblages was significantly associated with the specific habitat types and woody plant species present in the patches. The results suggest that neither the island biogeography theory nor the habitat diversity hypothesis can fully explain the impacts of habitat fragmentation on bird richness in our study system, with their roles primarily being linked to patch area. The findings that habitat and plant compositions were the major drivers of variation in bird assemblage composition offer valuable insights into urban planning and green initiatives. Conservation efforts should focus not only on preserving large areas, but also on preventing urban monocultures by promoting diverse habitats within those areas, contributing to the persistence of meta-communities.

## Introduction

Habitat fragmentation, driven by human land-use practices such as urbanization, agriculture, ranching, and logging, presents an extensive and pervasive threat to global biodiversity^1^. This process results in the creation of habitat patches that suffer from the combined negative effects of area reduction, isolation by distance, and boundary edge proliferation^1,2^. Generally, two competing frameworks—namely, the island biogeography theory^3^ and the habitat diversity hypothesis^4^—have been proposed to explain the adverse impacts of habitat fragmentation on biodiversity. Understanding these underlying mechanisms is an important prerequisite for informing sustainable land development, especially in urban landscapes that substantially fragment natural habitat^5–7^.

The application of the island biogeography theory^3^, originally formulated in the context of oceanic islands, to habitat fragmentation provides insights into the dynamics of species persistence and diversity in fragmented landscapes^8^. According to this theory, local extinctions of species become more frequent and stochastic as patch size decreases because populations of species in smaller patches are more susceptible to random events, environmental fluctuations, or catastrophic events. The immigration rates of species and rescue effects from other patches generally decrease as patches become more isolated, but the effects may be less pronounced for birds due to their highly developed flight capabilities^9,10^. Although the original island biogeography theory does not explicitly address edge effects, the concept becomes relevant when applied to fragmented habitats^11,12^. The conditions at the edges of a habitat patch, especially in areas subject to high human disturbance in urban environments, differ from those in the interior, negatively affecting species that are adapted to specific interior conditions^13^. As edge effects intensify with diminishing patch size, the core area of a patch—defined as the interior not influenced by edges—shrinks, contributing to a decline in biodiversity^11,14^. Nevertheless, edge effects can be variable^15–17^, and may be limited for birds, which can quickly evade threats and potentially habituate to human disturbance^18,19^. In some cases, bird species adapted to urban environments (i.e., urban adapters) may even benefit from edge ecotones, such as receiving supplemental food from human activities at patch edges^20^.

These landscape characteristics resulting from habitat fragmentation may sort species with specific functional traits^18,21,22^. For instance, larger-bodied birds such as the little egret *Egretta garzetta,* common cuckoo *Cuculus canorus*, and blue whistling thrush *Myophonus caeruleus* are more susceptible to urban-induced fragmentation than smaller-bodied species, as larger birds often require larger and more productive patches to meet their greater absolute energy requirements and support their territory ranges^22^. The trophic level of a species and its functional status in the community also relates strongly to its susceptibility to fragmentation. Carnivorous birds are generally more vulnerable to fragmentation^21,23^, whereas omnivorous birds tend to adapt well to fragmented environments, particularly those arising from urban urbanization^18^. Species with weaker dispersal abilities are generally less able to traverse the distance between suitable habitat patches and infiltrate communities than those with stronger dispersal abilities^23^. However, this limitation is likely less pronounced for birds, as they can easily fly between patches, except for territorial species that are reluctant to leave their nests during their breeding season^9,22^.

The habitat diversity hypothesis posits that area per se exerts minimal effects on species richness, with habitat diversity serving a key determinant^4,24^. Different species have specific habitat requirements, and smaller patches, which inherently contain fewer habitats, especially in urban environments, are expected to support fewer species^6,10^. In addition, a species’ habitat specificity or niche breadth can affect its occupancy and resilience in habitat fragments^25^. Specialists that prefer specific habitat types or plants are often more susceptible to habitat fragmentation than generalists^26^, especially during the breeding season when birds require specific habitats for nesting and food to raise broods^9^. This process primarily results in the selective disappearance of more habitat-specific species from smaller patches^22^. As a result, habitat nestedness drives species assemblage nestedness between patches (i.e., poor assemblages are subsets of richer ones)^27,28^. In contrast, substantial variation in habitats between patches may lead to species assemblage anti-nestedness, likely due to competitive interactions and resource partitioning^5,9,28,29^.

While some studies have presented conflicting findings regarding the relationship between patch area, habitat diversity, and their impact on species richness in fragmented habitats, recent suggestions propose that multiple processes operate simultaneously and in a mutually complementary manner to influence species richness^6,30^. However, the relative roles of such factors in regulating bird assemblages in fragmented habitats have not been fully explored. In particular, the potential significance of habitat composition as a major driver of variation in species assemblage composition across habitat fragments remains unresolved^9,26^. Such information is significant for informing landscape planning and habitat restoration strategies in fragmented landscapes with regard to conserving biodiversity, especially in the context of urban development^5,9,22^.

**Figure 1:**
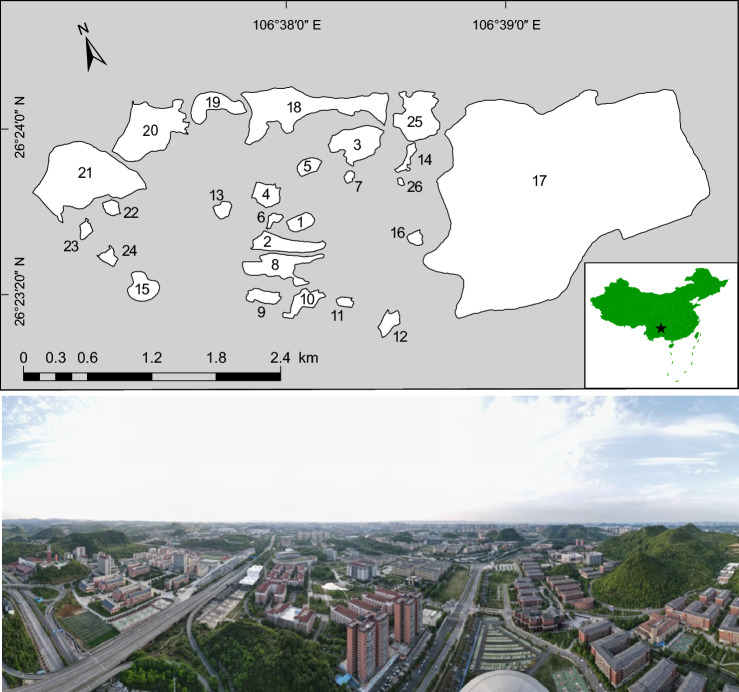
Map showing the distribution of 26 habitat patches (upper, generated by ArcGIS, version 10.7, https://www.esri.com/en-us/home) and landscape of Huaxi University Town, Guizhou, China (lower; source: Huahua Zhao).

In this study, we used birds, one of the most easily seen and well-known animal groups in urban environments and also are of significant global conservation interest, as the indicators to examine the roles of the habitat diversity hypothesis and the island biogeography theory in structuring species assemblages in 26 habitat patches over 20 km^2^ of urbanized landscape in southwest China (Fig. 1). These patches were once natural or semi-natural habitats that were originally part of the landscape but have since become fragmented due to urbanization. We investigated the effects of three important landscape characteristics [i.e., patch area, isolation, and perimeter to area ratio (PAR, a proxy for edge effects)^9,11^], habitat richness (the number of habitat types), and woody plant richness on bird richness, when discomposing direct from indirect effects. Furthermore, we examined how these characteristics sorted bird species according to their certain functional traits, including body size, hand-wing index (HWI, a proxy for flight efficiency in birds^31^), habitat specificity, clutch size, trophic level, territoriality, and flocking tendency. Finally, we examined how substantial differences in habitat type composition (including woodlands, scrublands, grasslands, wetlands, exposed karst rocks, and croplands) and woody plant species composition affected bird assemblage composition across the patches. This approach is aimed at improving landscape planning and habitat restoration strategies in fragmented landscapes particularly in the context of urban development, to conserve avian diversity.

## Results

### Effects of landscape and habitat characteristics on bird richness

We recorded a total of 70 breeding bird species, including songbirds, scansores, terrestrial birds, wading birds, and natatores (excluding birds recorded only once and fly-overs such as raptors, swallows and swifts; see Table S1 for more details), through 14 surveys. The number of species per patch varied from 14 to 56 (Table S2), with an average of 26 ± 10 (SD) species per patch. Among the five landscape and habitat characteristics measured, including patch area, isolation, PAR, habitat richness, and woody plant richness (for variations of these covariates across the patches, see Table S2), correlations were lower between patch area, isolation, and habitat richness (Fig. S1). Using linear regressions, we examined the relationships between bird richness and the three covariates with lower correlations. Among five models based on the island biogeography theory and the habitat diversity hypothesis, as well as the null model that included the intercept only, the best and well-fitted model suggested that patch area and habitat richness were important factors explaining the variation of bird richness (Table S3). We also employed linear regressions to examine the relationships between habitat richness with patch area and isolation (two models with combinations of the two covariates, as well as the null model), and between woody plant richness with patch area, isolation and habitat richness (five models with combinations of the three covariates, as well as the null model). The best and well-fitted model(s) suggested that patch area and isolation were important factors explaining the variation of habitat richness, while patch area, isolation, and habitat richness were important factors explaining the variation of woody plant richness (Table S3).

**Figure 2:**
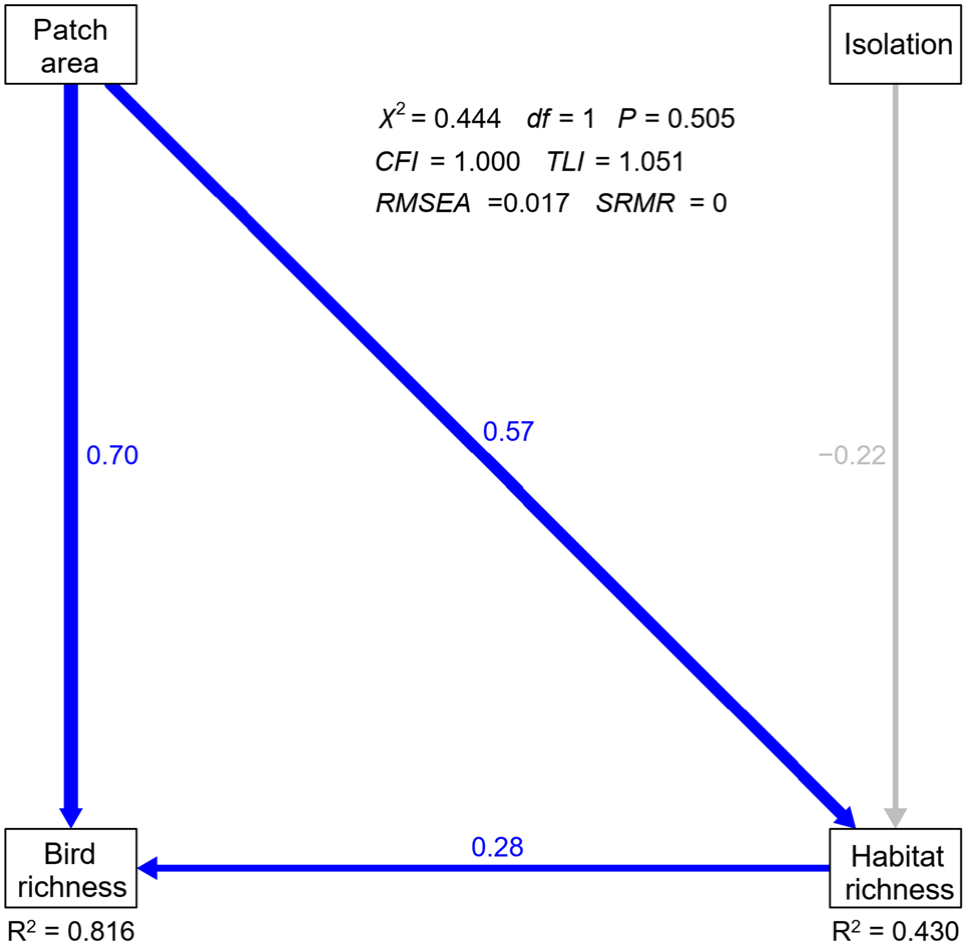
Summary of the best supported structural equation modeling (SEM) testing the direct and indirect effects of landscape and habitat characteristics on bird richness. Blue arrows represent significant positive paths, while gray arrows represent non-significant paths. Standardized path coefficient estimates are given next to each path. The proportion of explained variance (*R*^2^) for each endogenous variable, attributed to the effects of the other variables, is provided. Patch area was log-transformed, and all variables were centralized and standardized. Model goodness-of-fit was evaluated using chi-square test with statistics including chi-square (*χ*^2^), degrees of freedom (*df*), and P value (*P*), and four additional metrics, including comparative fit index (*CFI*), Tucker–Lewis index (*TLF*), root mean square error of approximation (*RMSEA*), and standard root mean square residual (*SRMR*).

We further used structural equation modeling (SEM) to examine causal relationships by decomposing direct from indirect effects, including the covariates in the best model(s), as well as those with high correlations with them but excluded in linear models. The best supported and well-fitted model derived from SEM included patch area, isolation, and habitat richness, while PAR and woody plant richness were not included in the model (Fig. 2). The model showed that patch area had a significant direct effect on bird richness, despite an indirect effect via habitat richness (Fig. 2), while isolation have no significant direct or indirect effects on bird richness.

### Effects of landscape and habitat characteristics on bird assemblage composition

**Figure 3:**
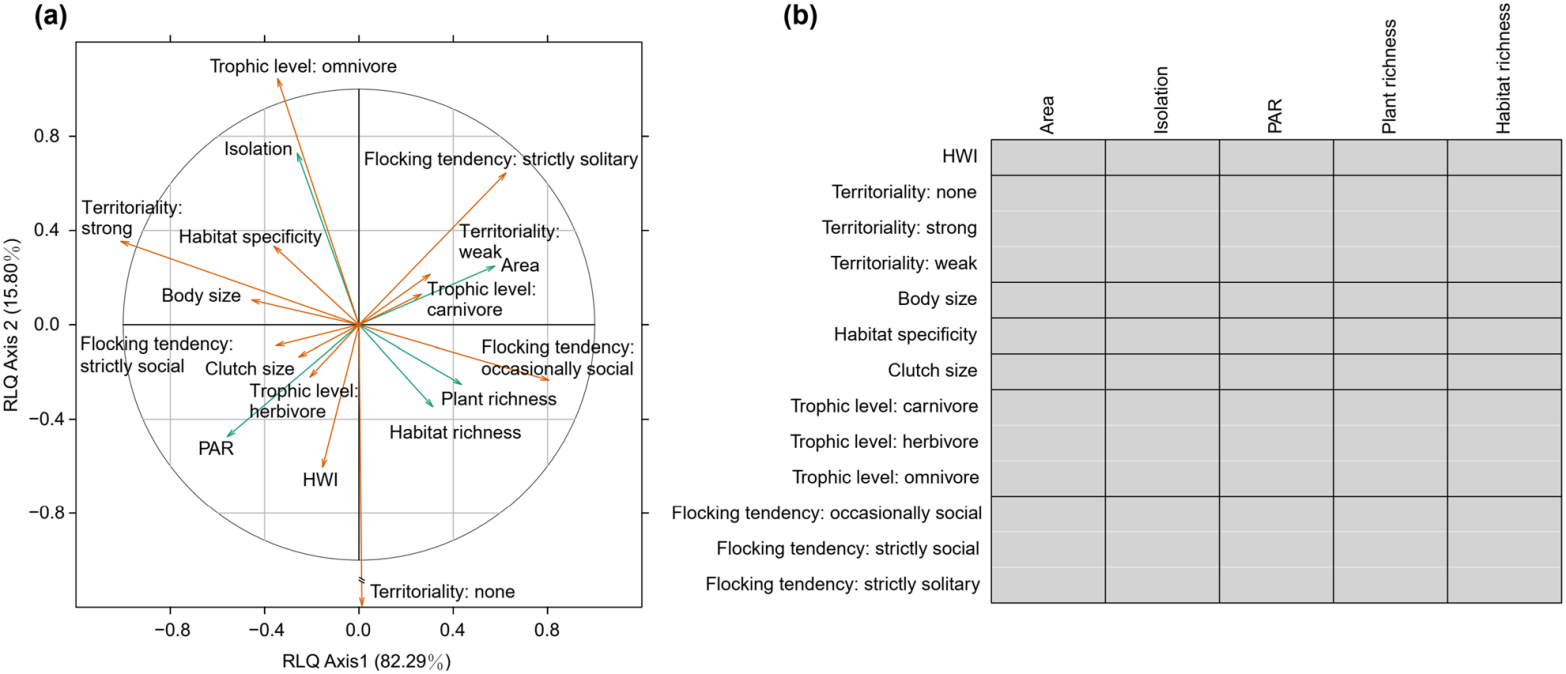
Results of r-mode linked to q-mode (RLQ) and fourth-corner analyses. (**a**) Correlation between landscape and habitat characteristics (green arrows) and bird species functional traits (orange arrows) along the first two RLQ axes, with the percentage of total co-inertia provided in parentheses. The correlation was mediated by abundance of bird species (i.e., bird assemblage composition). (**b**) Correlation table from the fourth-corner test displaying bivariate associations between landscape and habitat characteristics and bird species functional traits. Patch area and PAR were log-transformed. Cells shaded in gray indicate no significant association (for statistics, see Table S5).

We applied r-mode linked to q-mode (RLQ) analysis to examine covariation between the landscape and habitat characteristics, bird species functional traits (including body size, HWI, habitat specificity, clutch size, trophic level, territoriality and flocking tendency; for variations of these traits across species, see Table S4) by measuring abundance of bird species (i.e., bird assemblage composition). The first two RLQ axes accounted for 82.29% and 15.80% of total co-variation between the landscape and habitat characteristics of the patches and bird species functional traits mediated by bird assemblage composition. This corresponded to 33.82% with the correlation produced by the species abundance matrix correspondence analysis (L), and 97.73% and 52.73% with the potential inertia expressed for the first axis in principal component analysis of the landscape and habitat characteristics of the patches (R) and bird species functional traits (Q), respectively (Table 1). While the landscape and habitat characteristics and species functional traits correlated along the first two RLQ axes (Fig. 3a), fourth-corner analysis revealed no significant correlation between them (Fig. 3b).

**Table 1:** Results of r-mode linked to q-mode (RLQ) analysis of landscape and habitat characteristics of the patches (R), abundance of bird species (L), and bird species functional traits (Q).

	Axis 1 (%)	Axis 2 (%)
(a)		
R (PCA^1^)	3.41 (68.29)	0.91 (18.25)
L (CA^2^)	0.15 (19.60)	0.11 (15.03)
Q (PCA^1^)	2.13 (21.32)	1.95 (19.54)
(b)		
RLQ axis eigenvalues	0.06 (82.29)	0.01 (15.80)
Covariance	0.25	0.11
Correlation: L	0.13 (33.82)	0.10 (28.97)
Projected variance: R	3.34 (97.73)	4.17 (96.48)
Projected variance: Q	1.12 (52.73)	2.65 (64.91)

(a) Eigenvalues (% of total co-inertia) for the first two axes. (b) Summary of the RLQ analysis: eigenvalues (% of total co-inertia) for the first two RLQ axes, covariance and correlation (% variance) with the L matrix correspondence analysis and principal component analysis of projected variance (% variance) for the R and Q matrices. Patch area and PAR were log-transformed for analysis.

^1^Principal component analysis.

^2^Correspondence analysis.

**Figure 4:**
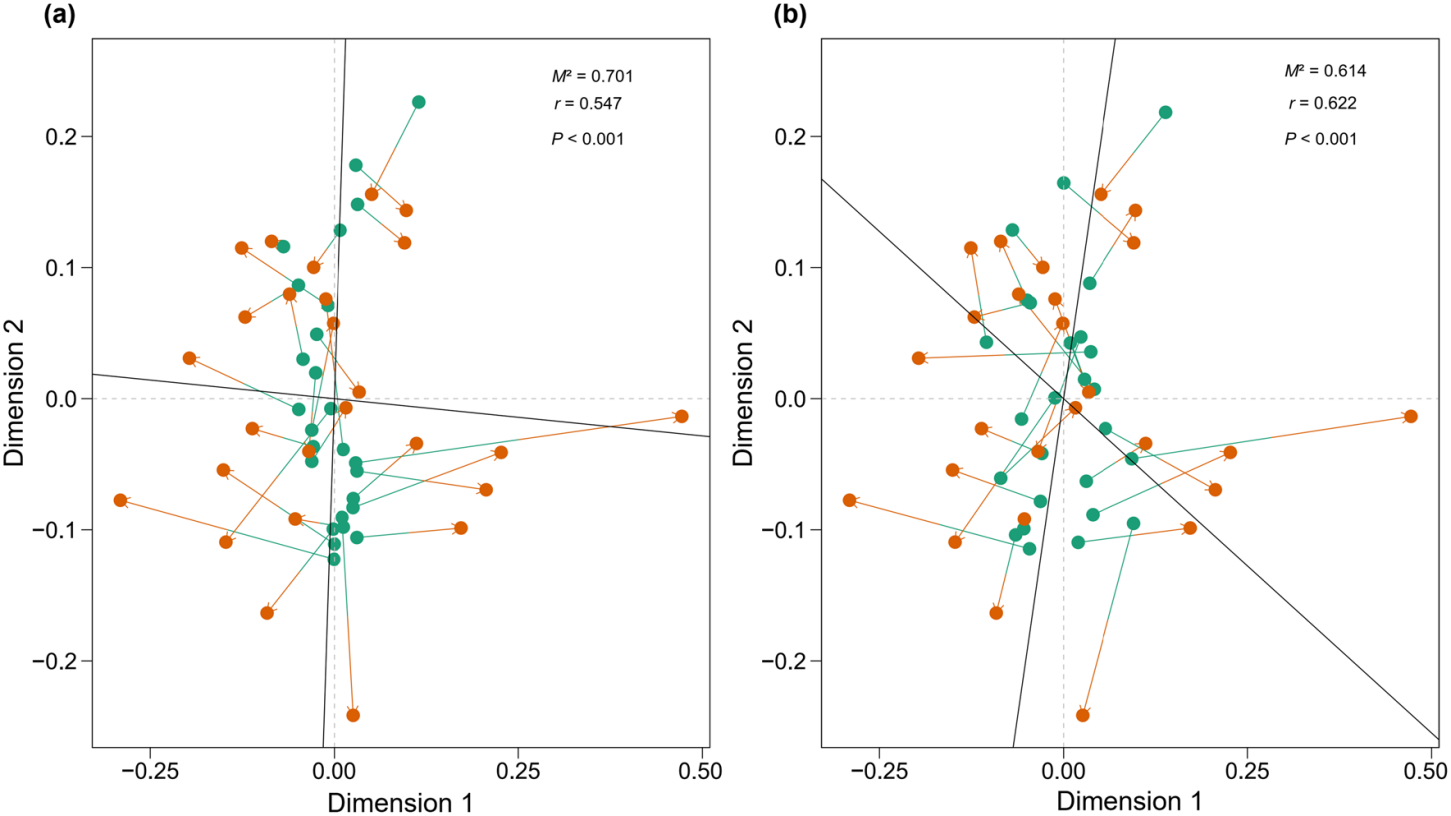
Results of Procrustes analysis based on a non-metric multidimensional scaling (NMDS) ordination. (**a**) Correlation between abundance of bird species (i.e., bird assemblage composition; green dots) and the presence/absence of habitat types (i.e., habitat type composition; orange dots); and (**b**) correlation between abundance of bird species (green dots) and the presence/absence of woody plant species (i.e., woody plant species composition; orange dots). The length of arrows connecting dots illustrates the difference in distance between the samples in the NMDS ordination space. A smaller sum of squares (*M*^2^) and a higher correlation coefficient (*r*) indicate a stronger correlation.

Furthermore, we performed Procrustes analysis to assess how the differences in bird assemblages across patches can be explained or linked to the compositional differences in habitat types and woody plant species. The results revealed a significant correlation between bird assemblage composition and habitat type composition per patch (Fig. 4a), and also between bird assemblage composition and woody plant species composition per patch (Fig. 4b).

## Discussion

Consistent with many studies on fragmented habitats^7,32^, our results showed a significant positive correlation between bird richness and patch area. Two competing theoretical frameworks have been proposed to explain the relationship. The island biogeography theory^3^ interprets that populations of species in smaller patches are more vulnerable to random events, environmental fluctuations, or catastrophic events, which could lead to local extinctions. In contrast, the habitat diversity hypothesis^4^ argues that patch area exerts minimal effects, with habitat diversity (measured as habitat richness and plant richness in our study) playing a crucial role in determining species richness. Our SEM results revealed that, as reported in several other habitat fragmentation systems^30,33^, patch area had direct and indirect (particularly through habitat richness) effects on bird richness. This suggests a complementary relationship between these two theoretical frameworks in explaining changes in bird richness in our study system.

While the island biogeography theory predicts that species richness decreases as isolation increases^3^, results from different studies are mixed^34^. Due to the highly developed flight capabilities of birds, short-distance isolation has limited effects on their dispersal or dispersion, potentially resulting in negligible isolation effects^9,10^. In our study area, there was relatively low isolation between different patches, with some patches being separated only by a single road. The fragmented patches were usually interspersed with artificial green spaces (e.g., flowerbeds and lawns) or areas cultivated with roadside trees, which may result in an influx of generalists or urban adapters that that gradually habituate to human disturbance^18,19,35^, such as the Eurasian tree sparrow *Passer montanus*, white-browed laughingthrush *Garrulax sannio*, and brown-breasted bulbul *Pycnonotus xanthorrhous*, thereby mitigating the level of isolation^9,20^.

We also found no significant correlation between bird richness and PAR, suggesting weak edge effects. Environmental conditions at the patch edges differ from those in the interior^13^. In habitats fragmented by urbanization, anthropogenic influences at the edge of habitat patches, such as traffic disturbance, noise pollution, and human interference, tend to be higher than in the patch interior^10,19^. Thus, patches with larger PAR (i.e., higher proportion of edge habitat) may be less suitable for species that are less tolerant of human disturbance^11,14^. However, there may be positive edge effects for some species^15–17^. For instance, some urban adapters that gradually habituate to human disturbance^18,19,35^, such as the Eurasian tree sparrow *Passer montanus*, white-browed laughingthrush *Garrulax sannio*, and brown-breasted bulbul *Pycnonotus xanthorrhous,* may benefit from patch edge ecotones, such as receiving supplemental food spillover from human activities at these edges^20^, leading to weak edge effects. In addition, the lack of significant edge effects may be due to extinction debt^36,37^, given that the urban landscape was established a relatively short period of 11‒14 years ago. The manifestation of effects related to the establishment of habitat edges necessitates a temporal progression^2^. Besides, the methods of data collection, which primarily involved transects running through the center of the patches and under-surveyed edge habitats, may have contributed to the absence of detected edge effects.

Although patch area had significant direct and indirect effects (mediated by habitat richness) on bird richness, these factors did not significantly sort bird species according to their specific functional traits. Some previous studies have suggested habitat fragmentation can indeed sort bird species with specific functional traits^18,21,22^. For example, Tai et al.^22^ demonstrated strong effects of trait-mediated environmental filters that selected bird species with smaller body mass and lower habitat specificity in 37 urban parks. Croci et al.^18^, using passerine data from 13 woodlands along a short rural–urban gradient, revealed that birds that adapted well to urban constrains such as habitat fragmentation tended to be omnivorous. We argue that the lack of sorting by landscape characteristics based on species functional traits in our study system may be attributed to extinction debt, where species may persist in smaller patches and experience a time lag before ultimately disappearing^2,36,37^.

Instead, we found a significant correlation between bird assemblage composition and habitat type composition (i.e., woodlands, scrublands, grasslands, wetlands, exposed karst rocks, and croplands), and also between bird assemblage composition and woody plant species composition across the patches. Given the substantial compositional variations in habitat types and woody plant species observed between the patches^38^, we argue that habitat heterogeneity provides opportunities for different species to occupy microhabitats across the patches, leading to the absence of a clear sorting by landscape characteristics based on species functional traits. Although birds generally have strong dispersal abilities, which would normally reduce the impact of isolation on dispersal limitations, intense interspecific competition during breeding, such as defending territories and nesting sites, may cause them to stay within their home range instead of dispersing between different patches^9^. For instance, we observed that birds such as the black drongo *Dicrurus macrocercus* and Red-billed blue magpie *Urocissa erythroryncha* evicting other species to protect their territories. Consequently, intense interspecific competition could result in species occupying different microhabitats within heterogeneous habitats, thus facilitating meta-community persistence^9,39^.

## Conclusion

Our results suggested that neither the island biogeography theory nor the habitat diversity hypothesis can work strongly in explaining the impacts of habitat fragmentation on bird richness in our study system. However, habitat type and plant compositions play a crucial role in driving the variation in bird assemblage composition across heterogeneous habitat patches. While further studies are needed to collect data, especially for woody plants, through the establishment of standard plots representative of the entire study patches, and to examine edge effects by comparing assemblages in sites with different distances to patch edges^16,40^, our findings already hold important implications for sustainable landscape planning and habitat restoration strategies. Given that habitat fragmentation due to urbanization is increasing in scale and rate, and that species richness will decrease with patch size reduction, we emphasize that conservation efforts in urban planning, construction and development should focus not only on preserving large areas but also on maintaining and promoting diverse habitats within those areas^22^. Habitats with more complex habitat types and greater plant species diversity not only help in preventing urban monocultures, as are common in many cities, especially in China^6,41^, but also favor meta-community persistence and contribute to the local pool of species^39^, potentially enhancing ecological resilience in urban environments.

## Materials and methods

### Study area

The study was conducted over 20 km^2^ in the central zone of Huaxi University Town, Guizhou province, southwest China (Fig. 1). The study area is a karst limestone plateau region at approximately 1200 m elevation, characterized by a subtropical humid temperate climate. Urban construction in this area commenced in 2009 to provide infrastructure for five universities that opened in 2012. Prior to this new development, the region comprised natural woodlots surrounded by croplands. Subsequently, human activity, mainly the construction of roads and buildings, transformed this region into a matrix of many small habitat patches. These habitat patches primarily consisted of woodlands, complemented by scrublands, grasslands, wetlands, exposed karst rocks, and croplands. For this study, we selected 26 habitat patches to survey birds (Fig. 1). In a recent survey, Cao^38^ described significant compositional variations in habitat types and woody plant species between these habitat patches.

### Field survey and data collection

We used ArcGIS 10.7 (www.esri.com) to outline the boundaries of the 26 patches, based on a Google-sourced base map. For each patch, we calculated three landscape characteristics (Table S2): area (ranging in size from 0.3 to 290.4 ha), isolation (Euclidean distance between the patch edge and the nearest patch edge, ranging from 10.8 to 273.3 m), and PAR (ranging from 27.0 to 657.0).

We surveyed birds using line transects. The line transects approximately crossed the center of each patch, while also covering different habitat types to maximize species detection. The total transect length in each patch was proportional to patch area (ranging from 82.5 to 7073.7 m; three transects established in the largest patch, and one single through other patches), based on Schoereder et al.^42^. We conducted surveys during the breeding season (April to August, inclusive) from 2017 to 2022, with observations made between 7:00‒10:00 a.m. and 4:00‒6:00 p.m. on weather-permitting days, excluding in rainy or foggy days. Each transect involved two collaborating observers walking at approximately 1.5 km/h, recording birds seen or heard within 50 m along both sides of the transect (see Zheng et al.^9^ for more details). Totally, we conducted 14 bird surveys in each patch, with three annually in 2017–2018, four in 2019, one in 2020, two in 2021, and one in 2022. We identified bird species based on MacKinnon et al.^43^ and Viney et al.^44^. We selected seven key functional traits for birds, including body size, HWI, habitat specificity, clutch size, trophic level, territoriality, and flocking tendency (see Table S4). These data were obtained in part from Zhao^45^ and published articles^31,46^, supplemented by our own direct observations (Table 2).

**Table 2:** Summary of bird species functional traits used.

Trait	Source	Scale	Description
Body size	Literature^45^	Continuous	Average individual bird body size (including males and females)
HWI	Literature^31^	Continuous	Hand-wing index, an estimate of wing shape used as a proxy for flight ability in birds
Habitat specificity	Literature^45^	Continuous	The sum of the species’ habitat type preference
Clutch size	Literature^45^	Continuous	The number of eggs produced during a single breeding event
Trophic level	Literature^45^	Categorical	Including three trophic categories: carnivores, herbivores, and omnivores
Territoriality	Literature^31,46^	Categorical	Exclusive, defended access to habitat and food resources throughout the year, including three levels: strong, weak, and none
Flocking tendency	Our observational data and Literature^45^	Categorical	The measurements of bird flocking behavior: strictly social, occasionally social or strictly solitary

Using Google imagery and surveys with binoculars along the same line-transects, Cao^38^ classified habitat types for each patch, including woodlands, scrublands, grasslands, wetlands, exposed karst rocks, or croplands. Furthermore, Cao^38^ recoded woody plant species occurring within 1 m (3 m for trees) on both sides of each transect, identifying them based on Editorial Committee of the Flora of China of Chinese Academy of Science^47^ and Editorial Committee of Higher Plants of China in Color^48^. We obtained the data on habitat types and woody plant species in each patch from Cao^38^. Habitat richness per patch ranged from one to five, and woody plant species richness ranged from 26 to 124 (see Table S2).

### Data analyses

We aggregated 14 surveys to assess bird assemblages, but excluded birds recorded only once and high-flying birds, including raptors, swallows, and swifts. Although we established the length of the transects in each patch roughly proportional to the patch area, the survey efforts were not directly proportional to area. We thus assessed the completeness of our survey for the largest and proportionally least sampled patch by creating the species rarefaction curve based on the number of individuals surveyed across the 6 years. The curve almost levelled off (Fig. S2), suggesting the sampling effort was almost sufficient on this and the smaller patches to capture the full breeding bird communities.

We tested the Spearman correlations between landscape and habitat characteristics (Fig. S1). For the linear regressions that examined the relationships between bird richness within the patches with their landscape and habitat characteristics, we only included covariates with lower correlations (i.e., patch area, habitat richness, and isolation) to avoid collinearity issues. We constructed five models using the three covariates based on the island biogeography theory and the habitat diversity hypothesis, along with the null model that included the intercept only. We calculated the Akaike information criterion corrected for small sample size (*AIC*c) for each model, and ranked the models based on their *AICc* values. The model with the lowest *AIC*c value was considered the best model, but these models with the difference in *AIC*c values (*ΔAIC*c) between the best model and them was less than or equal two had equivalent effects in explaining the data^49^.

Furthermore, we used the same approaches as those used to examine bird richness to investigate the relationships between habitat richness and patch area and isolation (two models with combinations of the two covariates, as well as the null model), and between woody plant richness and patch area, isolation and habitat richness (five models with combinations of the three covariates, as well as the null model). By including covariates in the best model(s), as well as those with high correlations with them but excluded in the linear models, we further used SEM to examine causal relationships by decomposing direct from indirect effects of these factors.

We pre-specified nine competing models (Fig. S3). Model 1 included direct effects of patch area and habitat richness on bird richness, with an indirect effect of patch area and isolation via habitat richness. Model 2 included direct effects of patch area and woody plant richness on bird richness with an indirect effect of patch area, isolation and habitat richness via woody plant richness, and an indirect effect of patch area and isolation on woody plant richness via habitat richness. Model 3 included direct effects of PAR and habitat richness on bird richness, with an indirect effect of patch area via PAR and an indirect effect of PAR and isolation via habitat richness. Model 4 included direct effects of PAR and woody plant richness on bird richness with an indirect effect of patch area via PAR and an indirect effect of PAR, isolation and habitat richness via woody plant richness, and an indirect effect of PAR and isolation on woody plant richness via habitat richness. Model 5 incorporated the additive effects of models 1 and 2, while model 6 incorporated the additive effects of models 3 and 4. Alternatively, model 7 incorporated the additive effects of models 1 and 3, while model 8 incorporated the additive effects of models 2 and 4. Finally, Model 9 incorporated the additive effects of models 5, 6, 7, and 8. We ranked these competing models using *AIC* values, considering the model with the lowest *AIC* value as the best supported. We evaluated model goodness-of-fit using chi-square test, and four additional metrics, including comparative fit index (*CFI*), Tucker–Lewis index (*TLF*), root mean square error of approximation (*RMSEA*), and standard root mean square residual (*SRMR*). A well-fitted model was assumed to have a *P* value > 0.05 for the chi-square test, *CFI* > 0.95, *TLF* > 0.90, *RMSEA* < 0.06, and *SRMR* < 0.09^50^.

For the RLQ analysis^51^ that examined covariation between the landscape and habitat characteristics and bird species functional traits, we produced three data matrices: landscape and habitat characteristics of each patch (R matrix), abundance of bird species per patch (L matrix), and functional traits per bird species (Q matrix). We carried out correspondence analysis for the L matrix, and principal component analysis for R and Q matrices. We calculated the ordination variance for each matrix separately. We summarized the joint structure of these RLQ analysis matrices over 49,999 permutations, to which we applied fourth-corner analysis^52^ to test the significance of correlations of the landscape and habitat characteristics with species functional traits according to the RLQ ordination axes^53^. Given multiple permutations, we adjusted *P* values using the false discovery rate (FDR) method^54^. As the correlations were not significant, further disentanglement of direct and indirect relationships was not pursued.

For the Procrustes analysis^55,56^ used to assess how the differences in bird assemblages across patches can be explained or linked to the compositional differences in habitat types and woody plant species, we included three data matrices: abundance of bird species (i.e., bird assemblage composition), presence/absence of habitat types (i.e., habitat type composition), and presence/absence of woody plant species (i.e., woody plant species composition) at each habitat patch. Firstly, we carried out a non-metric multidimensional scaling (NMDS) analysis (Bray–Curtis distance) for each of the matrices, to extract the coordinates of the eigenvalue axis for comparison. Subsequently, we applied the Procrustes analysis to measure the degree of congruence between pairs in the matrices. We used the sum of squares (*M*^2^) and *P* values after 49,999 permutations to evaluate the significance of these associations.

All analyses were performed in R 4.3.1^57^ using packages ade 4^58^, lavaan^59^ and vegan^60^. Unless otherwise stated, we report means ± SD.

### Supplementary Information

Supplementary Information.

## Data Availability

All data generated or analyzed during this study are included in this article (and its supplementary information files).
