# Supplementary material for: Quantifying the effects of landscape and habitat characteristics on structuring bird assemblages in urban habitat patches
Source: Sci Rep. 2024 Jun 3;14:12707. doi: 10.1038/s41598-024-63333-z (PMC11148024; doi:10.1038/s41598-024-63333-z)
Supplement: Supplementary file 1 — Supplementary Information. [file 41598_2024_63333_MOESM1_ESM.docx]

**Supplementary Information**

**Table S1.** Species-by-sites abundance matrix of breeding birds recorded on 26 habitat patches in Huaxi University Town, Guizhou, China. Excluded from the matrix are birds recorded only once and fly-overs, including raptors, swallows and swifts.

| Species | No. of patch | | | | | | | | | | | | | | | | | | | | | | | | | |
| --- | --- | --- | --- | --- | --- | --- | --- | --- | --- | --- | --- | --- | --- | --- | --- | --- | --- | --- | --- | --- | --- | --- | --- | --- | --- | --- |
|  | 1 | 2 | 3 | 4 | 5 | 6 | 7 | 8 | 9 | 10 | 11 | 12 | 13 | 14 | 15 | 16 | 17 | 18 | 19 | 20 | 21 | 22 | 23 | 24 | 25 | 26 |
| *Pycnonotus xanthorrhous* | 101 | 76 | 95 | 83 | 86 | 53 | 58 | 76 | 51 | 51 | 48 | 36 | 67 | 57 | 86 | 75 | 420 | 136 | 40 | 109 | 146 | 41 | 47 | 54 | 87 | 11 |
| *Garrulax sannio* | 92 | 90 | 94 | 47 | 38 | 36 | 45 | 53 | 61 | 41 | 28 | 21 | 81 | 22 | 54 | 47 | 191 | 64 | 28 | 49 | 87 | 20 | 29 | 23 | 50 | 11 |
| *Phoenicurus auroreus* | 8 | 17 | 30 | 11 | 7 | 19 | 13 | 10 | 15 | 12 | 7 | 10 | 15 | 10 | 15 | 26 | 104 | 39 | 8 | 18 | 27 | 20 | 23 | 7 | 20 | 8 |
| *Passer montanus* | 16 | 12 | 20 | 18 | 10 | 16 | 5 | 41 | 10 | 62 | 15 | 43 | 28 | 33 | 50 | 6 | 106 | 141 | 198 | 60 | 90 | 76 | 62 | 25 | 140 | 13 |
| *Motacilla alba* | 7 | 9 | 10 | 2 | 9 | 2 | 11 | 22 | 6 | 13 | 4 | 4 | 8 | 10 | 7 | 28 | 30 | 10 | 5 | 14 | 19 | 10 | 8 | 8 | 13 | 7 |
| *Spizixos semitorques* | 23 | 31 | 11 | 21 | 6 | 7 | 11 | 44 | 16 | 19 | 12 | 18 | 25 | 15 | 25 | 21 | 111 | 62 | 27 | 43 | 89 | 4 | 11 | 7 | 73 | 7 |
| *Streptopelia orientalis* | 6 | 12 | 4 | 3 | 1 | 7 | 7 | 3 | 13 | 3 | 4 | 3 | 3 | 6 | 8 | 6 | 15 | 5 | 12 | 6 | 8 | 0 | 0 | 2 | 20 | 3 |
| *Urocissa erythrorhyncha* | 5 | 10 | 6 | 14 | 2 | 6 | 3 | 13 | 10 | 10 | 5 | 18 | 9 | 9 | 7 | 32 | 26 | 7 | 6 | 3 | 28 | 1 | 2 | 0 | 12 | 21 |
| *Parus cinereus* | 18 | 38 | 14 | 4 | 4 | 11 | 7 | 29 | 8 | 21 | 0 | 1 | 10 | 15 | 5 | 9 | 49 | 10 | 5 | 8 | 8 | 1 | 3 | 0 | 21 | 7 |
| *Chloris sinica* | 5 | 27 | 22 | 11 | 10 | 12 | 0 | 2 | 16 | 21 | 3 | 5 | 5 | 11 | 10 | 0 | 42 | 38 | 2 | 15 | 25 | 3 | 23 | 9 | 24 | 1 |
| *Pica pica* | 0 | 11 | 7 | 7 | 1 | 12 | 0 | 6 | 7 | 3 | 0 | 3 | 8 | 3 | 29 | 0 | 20 | 19 | 6 | 7 | 9 | 8 | 7 | 9 | 8 | 10 |
| *Lanius schach* | 0 | 3 | 3 | 2 | 0 | 3 | 3 | 1 | 1 | 1 | 1 | 8 | 2 | 1 | 5 | 2 | 22 | 9 | 0 | 4 | 4 | 1 | 3 | 9 | 4 | 4 |
| *Horornis fortipes* | 1 | 1 | 11 | 3 | 6 | 0 | 0 | 3 | 1 | 1 | 0 | 2 | 1 | 0 | 4 | 7 | 115 | 10 | 0 | 23 | 36 | 2 | 8 | 2 | 9 | 0 |
| *Copsychus saularis* | 3 | 1 | 2 | 1 | 1 | 0 | 3 | 1 | 5 | 4 | 0 | 3 | 3 | 11 | 2 | 3 | 8 | 11 | 15 | 1 | 3 | 0 | 0 | 0 | 12 | 1 |
| *Zosterops japonicus* | 16 | 19 | 10 | 17 | 2 | 2 | 0 | 11 | 10 | 0 | 0 | 2 | 10 | 2 | 0 | 4 | 62 | 8 | 5 | 11 | 5 | 2 | 0 | 0 | 14 | 10 |
| *Aegithalos concinnus* | 3 | 31 | 24 | 0 | 0 | 1 | 0 | 0 | 6 | 6 | 0 | 0 | 17 | 1 | 0 | 5 | 48 | 35 | 0 | 6 | 3 | 0 | 0 | 0 | 4 | 0 |
| *Acridotheres cristatellus* | 0 | 0 | 1 | 0 | 0 | 0 | 0 | 5 | 0 | 0 | 0 | 2 | 0 | 0 | 3 | 1 | 16 | 12 | 2 | 17 | 10 | 0 | 1 | 3 | 8 | 0 |
| *Phylloscopus proregulus* | 11 | 17 | 10 | 2 | 2 | 8 | 7 | 2 | 1 | 0 | 0 | 0 | 0 | 1 | 3 | 0 | 19 | 0 | 2 | 4 | 0 | 0 | 0 | 0 | 3 | 0 |
| *Abroscopus albogularis* | 1 | 7 | 1 | 1 | 0 | 0 | 0 | 4 | 1 | 1 | 0 | 0 | 0 | 2 | 0 | 2 | 9 | 2 | 3 | 2 | 1 | 0 | 0 | 0 | 6 | 0 |
| *Prinia inornata* | 0 | 0 | 0 | 0 | 0 | 0 | 0 | 0 | 0 | 0 | 1 | 1 | 0 | 0 | 8 | 0 | 10 | 2 | 0 | 4 | 1 | 5 | 2 | 10 | 0 | 0 |
| *Lanius tigrinus* | 0 | 0 | 0 | 0 | 0 | 1 | 0 | 6 | 1 | 0 | 0 | 0 | 0 | 1 | 0 | 0 | 1 | 0 | 6 | 0 | 0 | 0 | 0 | 1 | 10 | 0 |
| *Turdus mandarinus* | 0 | 0 | 2 | 0 | 0 | 0 | 1 | 0 | 3 | 0 | 0 | 2 | 0 | 4 | 0 | 4 | 3 | 1 | 4 | 5 | 0 | 0 | 0 | 0 | 22 | 2 |
| *Streptopelia chinensis* | 1 | 6 | 1 | 0 | 0 | 0 | 0 | 0 | 0 | 0 | 0 | 1 | 1 | 0 | 0 | 0 | 2 | 3 | 15 | 8 | 2 | 0 | 0 | 0 | 3 | 0 |
| *Lanius cristatus* | 1 | 0 | 0 | 0 | 0 | 0 | 0 | 3 | 0 | 0 | 0 | 0 | 2 | 5 | 0 | 0 | 1 | 0 | 0 | 0 | 1 | 0 | 0 | 1 | 4 | 0 |
| *Saxicola maurus* | 0 | 0 | 0 | 0 | 0 | 0 | 0 | 1 | 0 | 0 | 0 | 0 | 0 | 0 | 21 | 0 | 24 | 0 | 0 | 8 | 8 | 7 | 11 | 16 | 0 | 0 |
| *Lonchura striata* | 0 | 0 | 0 | 0 | 0 | 0 | 0 | 0 | 2 | 0 | 0 | 0 | 0 | 0 | 0 | 0 | 1 | 0 | 1 | 1 | 2 | 2 | 0 | 1 | 0 | 0 |
| *Eophona migratoria* | 0 | 0 | 1 | 0 | 0 | 0 | 0 | 4 | 9 | 0 | 0 | 0 | 0 | 0 | 0 | 2 | 2 | 2 | 1 | 0 | 0 | 0 | 0 | 0 | 18 | 3 |
| *Eudynamys scolopaceus* | 0 | 0 | 4 | 0 | 0 | 0 | 0 | 0 | 1 | 0 | 0 | 0 | 0 | 4 | 0 | 0 | 2 | 4 | 0 | 1 | 1 | 0 | 0 | 0 | 12 | 2 |
| *Pericrocotus roseus* | 0 | 3 | 0 | 0 | 0 | 0 | 0 | 4 | 0 | 4 | 4 | 0 | 0 | 0 | 0 | 0 | 1 | 0 | 1 | 0 | 0 | 0 | 0 | 0 | 6 | 0 |
| *Turdus dissimilis* | 0 | 0 | 0 | 0 | 0 | 0 | 0 | 4 | 15 | 1 | 0 | 2 | 2 | 2 | 0 | 4 | 1 | 1 | 4 | 1 | 0 | 0 | 0 | 0 | 28 | 4 |
| *Monticola solitarius* | 4 | 0 | 4 | 0 | 0 | 0 | 0 | 2 | 0 | 0 | 0 | 1 | 0 | 0 | 0 | 0 | 0 | 3 | 2 | 0 | 0 | 0 | 6 | 0 | 2 | 0 |
| *Myophonus caeruleus* | 0 | 0 | 4 | 0 | 0 | 0 | 0 | 0 | 0 | 0 | 0 | 0 | 0 | 0 | 0 | 0 | 12 | 2 | 0 | 3 | 5 | 0 | 0 | 0 | 5 | 0 |
| *Schoeniparus dubius* | 5 | 8 | 8 | 5 | 0 | 1 | 1 | 2 | 1 | 3 | 1 | 0 | 0 | 0 | 0 | 3 | 7 | 0 | 0 | 1 | 2 | 0 | 0 | 0 | 2 | 0 |
| *Passer cinnamomeus* | 0 | 0 | 0 | 0 | 0 | 0 | 0 | 0 | 10 | 2 | 0 | 0 | 0 | 4 | 0 | 6 | 7 | 0 | 1 | 0 | 0 | 0 | 0 | 0 | 8 | 0 |
| *Motacilla cinerea* | 0 | 0 | 0 | 0 | 0 | 0 | 0 | 0 | 0 | 0 | 0 | 0 | 0 | 0 | 0 | 1 | 1 | 0 | 1 | 2 | 2 | 0 | 0 | 0 | 0 | 0 |
| *Erythrogenys gravivox* | 0 | 0 | 0 | 0 | 0 | 0 | 0 | 0 | 0 | 0 | 0 | 0 | 0 | 2 | 0 | 1 | 28 | 2 | 0 | 2 | 7 | 0 | 0 | 3 | 0 | 0 |
| *Sinosuthora alphonsiana* | 3 | 0 | 3 | 0 | 0 | 0 | 0 | 0 | 0 | 0 | 0 | 0 | 0 | 1 | 0 | 0 | 25 | 0 | 1 | 0 | 1 | 0 | 0 | 0 | 7 | 0 |
| *Prinia crinigera* | 0 | 0 | 0 | 0 | 0 | 0 | 0 | 0 | 0 | 0 | 1 | 0 | 0 | 0 | 1 | 0 | 12 | 5 | 0 | 4 | 2 | 1 | 1 | 0 | 0 | 0 |
| *Lonchura punctulata* | 0 | 0 | 0 | 0 | 0 | 0 | 2 | 0 | 0 | 4 | 0 | 13 | 0 | 0 | 1 | 0 | 6 | 4 | 0 | 11 | 13 | 0 | 0 | 0 | 0 | 0 |
| *Cyanoderma ruficeps* | 0 | 0 | 1 | 0 | 0 | 0 | 0 | 0 | 0 | 0 | 0 | 0 | 0 | 1 | 0 | 2 | 20 | 1 | 0 | 4 | 1 | 0 | 0 | 0 | 1 | 1 |
| *Spodiopsar sericeus* | 0 | 0 | 0 | 0 | 0 | 0 | 1 | 0 | 0 | 0 | 0 | 0 | 0 | 0 | 0 | 1 | 5 | 0 | 4 | 1 | 0 | 0 | 0 | 0 | 5 | 0 |
| *Terpsiphone incei* | 1 | 0 | 0 | 0 | 0 | 0 | 0 | 0 | 0 | 0 | 0 | 0 | 0 | 0 | 0 | 0 | 0 | 0 | 1 | 0 | 0 | 0 | 0 | 0 | 0 | 0 |
| *Pomatorhinus ruficollis* | 0 | 0 | 1 | 0 | 0 | 0 | 0 | 0 | 0 | 0 | 0 | 0 | 0 | 0 | 0 | 4 | 16 | 1 | 0 | 0 | 0 | 0 | 0 | 0 | 0 | 0 |
| *Emberiza elegans* | 0 | 0 | 0 | 0 | 0 | 0 | 0 | 0 | 0 | 0 | 0 | 0 | 0 | 0 | 0 | 0 | 5 | 1 | 0 | 4 | 0 | 0 | 0 | 0 | 0 | 0 |
| *Dicrurus hottentottus* | 0 | 3 | 0 | 0 | 0 | 0 | 0 | 0 | 0 | 0 | 0 | 0 | 0 | 0 | 0 | 0 | 0 | 0 | 1 | 0 | 0 | 0 | 0 | 0 | 1 | 0 |
| *Garrulus glandarius* | 0 | 0 | 0 | 0 | 0 | 0 | 0 | 0 | 0 | 0 | 0 | 0 | 0 | 5 | 0 | 0 | 3 | 0 | 0 | 0 | 0 | 0 | 0 | 0 | 0 | 2 |
| *Eumyias thalassinus* | 1 | 0 | 0 | 0 | 0 | 0 | 0 | 0 | 2 | 0 | 0 | 0 | 0 | 1 | 0 | 0 | 3 | 1 | 2 | 0 | 0 | 0 | 0 | 1 | 7 | 0 |
| *Muscicapa dauurica* | 0 | 0 | 0 | 0 | 0 | 0 | 0 | 0 | 0 | 0 | 0 | 0 | 1 | 0 | 0 | 0 | 0 | 0 | 0 | 0 | 0 | 0 | 0 | 0 | 1 | 0 |
| *Yuhina castaniceps* | 0 | 0 | 8 | 0 | 0 | 0 | 0 | 0 | 0 | 5 | 0 | 0 | 0 | 0 | 0 | 0 | 15 | 0 | 0 | 0 | 0 | 0 | 0 | 0 | 0 | 0 |
| *Emberiza godlewskii* | 0 | 0 | 1 | 0 | 0 | 0 | 0 | 0 | 0 | 0 | 0 | 0 | 0 | 0 | 0 | 0 | 1 | 0 | 0 | 0 | 0 | 0 | 0 | 0 | 0 | 0 |
| *Phasianus colchicus* | 0 | 0 | 0 | 0 | 0 | 0 | 0 | 0 | 0 | 0 | 0 | 0 | 0 | 0 | 2 | 0 | 0 | 0 | 0 | 1 | 0 | 0 | 0 | 0 | 0 | 0 |
| *Emberiza fucata* | 0 | 0 | 0 | 0 | 0 | 0 | 0 | 0 | 0 | 0 | 0 | 0 | 0 | 0 | 3 | 0 | 1 | 0 | 0 | 0 | 0 | 0 | 0 | 0 | 0 | 0 |
| *Psilopogon virens* | 0 | 0 | 0 | 0 | 0 | 0 | 0 | 0 | 0 | 0 | 0 | 0 | 0 | 0 | 0 | 0 | 0 | 0 | 0 | 0 | 0 | 0 | 0 | 0 | 4 | 0 |
| *Picus canus* | 0 | 0 | 0 | 0 | 0 | 0 | 0 | 0 | 0 | 0 | 0 | 0 | 0 | 0 | 0 | 0 | 0 | 0 | 0 | 0 | 0 | 0 | 0 | 0 | 2 | 0 |
| *Pericrocotus cantonensis* | 0 | 0 | 0 | 0 | 0 | 0 | 0 | 5 | 0 | 0 | 0 | 0 | 0 | 0 | 0 | 0 | 0 | 0 | 1 | 0 | 0 | 0 | 0 | 0 | 0 | 0 |
| *Emberiza cioides* | 0 | 0 | 0 | 0 | 0 | 0 | 0 | 0 | 0 | 0 | 0 | 0 | 0 | 0 | 0 | 0 | 1 | 1 | 0 | 0 | 0 | 0 | 0 | 0 | 0 | 0 |
| *Culicicapa ceylonensis* | 0 | 3 | 0 | 0 | 0 | 0 | 0 | 0 | 0 | 0 | 0 | 0 | 0 | 0 | 0 | 0 | 0 | 0 | 0 | 0 | 0 | 0 | 0 | 0 | 2 | 0 |
| *Phylloscopus trochiloides* | 0 | 0 | 0 | 0 | 0 | 0 | 0 | 0 | 2 | 0 | 0 | 0 | 0 | 0 | 0 | 0 | 0 | 0 | 0 | 0 | 0 | 0 | 0 | 0 | 1 | 0 |
| *Sinosuthora webbiana* | 2 | 0 | 0 | 0 | 0 | 0 | 0 | 0 | 0 | 0 | 0 | 0 | 0 | 0 | 0 | 0 | 1 | 2 | 0 | 0 | 3 | 0 | 0 | 0 | 0 | 0 |
| *Hierococcyx sparverioides* | 0 | 0 | 0 | 0 | 0 | 0 | 0 | 0 | 0 | 1 | 0 | 0 | 0 | 0 | 0 | 0 | 2 | 0 | 1 | 0 | 0 | 0 | 0 | 0 | 0 | 0 |
| *Egretta garzetta* | 0 | 0 | 0 | 0 | 0 | 0 | 0 | 0 | 1 | 0 | 0 | 0 | 0 | 1 | 0 | 1 | 8 | 1 | 0 | 2 | 18 | 0 | 0 | 0 | 1 | 0 |
| *Rhyacornis fuliginosa* | 0 | 0 | 0 | 0 | 0 | 0 | 0 | 0 | 0 | 0 | 0 | 0 | 0 | 0 | 0 | 0 | 20 | 6 | 0 | 5 | 15 | 0 | 0 | 0 | 9 | 0 |
| *Ardeola bacchus* | 0 | 0 | 0 | 0 | 0 | 0 | 0 | 0 | 0 | 0 | 0 | 0 | 0 | 0 | 0 | 2 | 10 | 1 | 0 | 5 | 16 | 0 | 0 | 0 | 1 | 0 |
| *Zapornia akool* | 0 | 0 | 0 | 0 | 0 | 0 | 0 | 0 | 0 | 0 | 0 | 0 | 0 | 0 | 0 | 0 | 0 | 0 | 0 | 2 | 1 | 0 | 0 | 0 | 0 | 0 |
| *Tachybaptus ruficollis* | 0 | 0 | 0 | 0 | 0 | 0 | 0 | 0 | 0 | 0 | 0 | 0 | 0 | 0 | 0 | 0 | 7 | 0 | 0 | 0 | 0 | 0 | 0 | 0 | 0 | 0 |
| *Alcedo atthis* | 0 | 0 | 0 | 0 | 0 | 0 | 0 | 0 | 0 | 0 | 0 | 0 | 0 | 0 | 0 | 0 | 0 | 0 | 0 | 0 | 7 | 0 | 0 | 0 | 0 | 0 |
| *Chaimarrornis leucocephalus* | 0 | 0 | 0 | 3 | 0 | 0 | 0 | 0 | 0 | 0 | 0 | 0 | 0 | 0 | 0 | 0 | 0 | 1 | 0 | 0 | 0 | 0 | 0 | 0 | 0 | 0 |
| *Picumnus innominatus* | 0 | 0 | 1 | 0 | 0 | 0 | 0 | 1 | 0 | 0 | 0 | 0 | 0 | 2 | 0 | 0 | 1 | 0 | 0 | 0 | 0 | 0 | 0 | 0 | 0 | 0 |
| *Leiothrix lutea* | 0 | 0 | 0 | 0 | 0 | 0 | 0 | 0 | 0 | 0 | 0 | 0 | 0 | 0 | 0 | 0 | 5 | 0 | 0 | 0 | 0 | 0 | 0 | 0 | 0 | 0 |
| *Ardea cinerea* | 0 | 0 | 2 | 0 | 0 | 0 | 0 | 0 | 0 | 0 | 0 | 0 | 0 | 0 | 0 | 0 | 0 | 0 | 0 | 0 | 4 | 0 | 0 | 0 | 0 | 0 |

**Table S2.** Landscape and habitat characteristics of 26 habitat patches and line-transects established in each patch in Huaxi University Town, Guizhou, China.

| No. of patch | Area  (ha) | Isolation (m) | PAR | Woody plant richness | Habitat richness | Number of line- transects | Total length of line- transects (m) |
| --- | --- | --- | --- | --- | --- | --- | --- |
| 1 | 2.4 | 44.2 | 256.0 | 60 | 3 | 4 | 442.6 |
| 2 | 6.2 | 20.0 | 229.1 | 96 | 2 | 3 | 915.5 |
| 3 | 9.4 | 43.1 | 145.8 | 72 | 3 | 2 | 926.3 |
| 4 | 3.6 | 56.5 | 217.2 | 51 | 2 | 1 | 336.3 |
| 5 | 2.1 | 110.3 | 277.5 | 39 | 1 | 1 | 298.6 |
| 6 | 0.9 | 23.4 | 510.2 | 53 | 3 | 1 | 172.1 |
| 7 | 0.6 | 43.1 | 523.7 | 39 | 2 | 1 | 126.2 |
| 8 | 8.6 | 20.0 | 235.3 | 97 | 2 | 1 | 617.1 |
| 9 | 2.6 | 97.4 | 291.0 | 80 | 3 | 1 | 282.3 |
| 10 | 3.7 | 33.3 | 287.5 | 89 | 3 | 2 | 392.8 |
| 11 | 0.9 | 94.5 | 454.9 | 60 | 2 | 1 | 153.6 |
| 12 | 2.3 | 273.3 | 298.9 | 69 | 2 | 2 | 293.2 |
| 13 | 1.5 | 180.5 | 320.4 | 55 | 2 | 1 | 250.9 |
| 14 | 1.5 | 10.8 | 512.4 | 62 | 3 | 2 | 254.1 |
| 15 | 3.2 | 261.9 | 219.9 | 29 | 2 | 1 | 252.6 |
| 16 | 1.2 | 91.2 | 357.2 | 79 | 3 | 2 | 159.1 |
| 17 | 290.4 | 56.5 | 27.0 | 124 | 4 | 7 | 7073.7 |
| 18 | 25.5 | 37.4 | 141.2 | 102 | 5 | 3 | 1302.8 |
| 19 | 6.5 | 33.8 | 197.5 | 111 | 4 | 1 | 558.6 |
| 20 | 19.0 | 33.8 | 118.9 | 72 | 5 | 1 | 892.2 |
| 21 | 34.2 | 20.4 | 77.9 | 87 | 4 | 2 | 1064.4 |
| 22 | 1.4 | 20.4 | 334.0 | 25 | 1 | 1 | 183.2 |
| 23 | 1.1 | 85.1 | 438.1 | 37 | 1 | 1 | 204.5 |
| 24 | 1.4 | 174.5 | 396.5 | 31 | 1 | 1 | 244.3 |
| 25 | 11.5 | 10.8 | 141.6 | 85 | 2 | 2 | 585.4 |
| 26 | 0.3 | 47.7 | 657.0 | 48 | 2 | 2 | 82.5 |

**Table S3.** The best models in linear regressions examining the relationships between bird richness and patch area, habitat richness, and isolation, between habitat richness and patch area and isolation, and between woody plant richness and patch area, isolation and habitat richness. Patch area was log-transformed, and all variables were centralized and standardized. The statistics include coefficient of determination (*R*^2^), number of parameters (*K*), log-likelihood value (*logLik*), Akaike’s information criterion corrected for small sample size (*AIC_c_*), difference in *AIC_c_* relative to the minimum *AIC_c_* (Δ*AIC_c_*), and Akaike weights (*w_i_*).

| Model | *R*^2^ | *K* | *logLik* | *AIC_c_* | Δ*AIC_c_* | *w_i_* |
| --- | --- | --- | --- | --- | --- | --- |
| *Bird richness* |  |  |  |  |  |  |
| Patch area+ Habitat richness | 0.77 | 4 | -14.40 | 38.70 | 0 | 0.65 |
| Patch area + Isolation + Habitat richness | 0.78 | 5 | -14.17 | 41.30 | 2.65 | 0.17 |
| Patch area | 0.51 | 3 | -17.48 | 42.00 | 3.35 | 0.12 |
| Patch area + Isolation | 0.82 | 4 | -16.74 | 43.40 | 4.68 | 0.06 |
| Habitat richness | 0.82 | 3 | -26.98 | 61.10 | 22.36 | 0 |
| Null | 0 | 2 | -36.38 | 77.30 | 38.59 | 0 |
| *Habitat richness* |  |  |  |  |  |  |
| Patch area | 0.39 | 3 | -30.06 | 67.20 | 0 | 0.60 |
| Patch area + Isolation | 0.43 | 4 | -29.07 | 68.00 | 0.83 | 0.40 |
| Null | 0 | 2 | -36.38 | 77.30 | 10.07 | 0 |
| *Plant richness* |  |  |  |  |  |  |
| Patch area + Habitat richness | 0.54 | 4 | -23.75 | 57.40 | 0 | 0.49 |
| Patch area + Isolation + Habitat richness | 0.58 | 5 | -23.07 | 59.10 | 1.74 | 0.21 |
| Patch area | 0.47 | 3 | -26.39 | 59.90 | 2.47 | 0.14 |
| Patch area + Isolation | 0.62 | 4 | -25.05 | 60.00 | 2.62 | 0.13 |
| Habitat richness | 0.64 | 3 | -28.20 | 63.50 | 6.09 | 0.02 |
| Null | 0 | 2 | -36.38 | 77.30 | 19.89 | 0 |

**Table S4.** Seven key functional traits of bird species.

| Species | HWI | Territoriality | Body size (cm) | Habitat specificity | Clutch size | Trophic level | Flocking tendency |
| --- | --- | --- | --- | --- | --- | --- | --- |
| *Pycnonotus xanthorrhous* | 15.81 | weak | 194 | 6 | 3.5 | herbivore | strictly social |
| *Garrulax sannio* | 8.08 | strong | 224.25 | 5 | 4 | carnivore | strictly social |
| *Phoenicurus auroreus* | 20.53 | weak | 142.75 | 5 | 6.5 | carnivore | strictly solitary |
| *Passer montanus* | 22.84 | none | 132 | 5 | 5.5 | herbivore | strictly social |
| *Motacilla alba* | 30.98 | weak | 175.75 | 3 | 5.5 | carnivore | strictly solitary |
| *Spizixos semitorques* | 14.85 | weak | 191.75 | 4 | 3.5 | herbivore | occasionally social |
| *Streptopelia orientalis* | 32.75 | weak | 314.75 | 4 | 2 | herbivore | occasionally social |
| *Urocissa erythrorhyncha* | 19.62 | strong | 580.75 | 4 | 4.5 | carnivore | strictly social |
| *Parus cinereus* | 17.00 | weak | 134.25 | 6 | 7.5 | carnivore | strictly social |
| *Chloris sinica* | 33.54 | weak | 130 | 4 | 4.5 | herbivore | occasionally social |
| *Pica pica* | 21.69 | weak | 422.5 | 4 | 6.5 | omnivore | strictly social |
| *Lanius schach* | 20.00 | weak | 248.5 | 6 | 4.5 | carnivore | strictly solitary |
| *Horornis fortipes* | 11.26 | weak | 114 | 5 | 4 | carnivore | strictly solitary |
| *Copsychus saularis* | 18.59 | strong | 201.5 | 4 | 5 | carnivore | strictly solitary |
| *Zosterops japonicus* | 21.80 | weak | 103.25 | 5 | 3 | omnivore | strictly social |
| *Aegithalos concinnus* | 17.63 | weak | 100.75 | 4 | 6.5 | carnivore | strictly social |
| *Acridotheres cristatellus* | 23.70 | none | 242.25 | 4 | 4.5 | carnivore | strictly social |
| *Phylloscopus proregulus* | 18.85 | weak | 94.25 | 3 | 5.5 | carnivore | strictly social |
| *Abroscopus albogularis* | 13.26 | weak | 94.5 | 2 | 4.5 | carnivore | strictly social |
| *Prinia inornata* | 14.17 | strong | 132 | 3 | 4.93 | carnivore | occasionally social |
| *Lanius tigrinus* | 29.24 | weak | 170.75 | 3 | 5.5 | carnivore | strictly solitary |
| *Turdus mandarinus* | 24.06 | weak | 258 | 5 | 5 | carnivore | occasionally social |
| *Streptopelia chinensis* | 30.61 | weak | 304.25 | 4 | 2 | herbivore | strictly social |
| *Lanius cristatus* | 26.96 | weak | 189.25 | 5 | 6 | carnivore | strictly solitary |
| *Saxicola maurus* | 19.90 | weak | 129.75 | 5 | 6.5 | carnivore | strictly solitary |
| *Lonchura striata* | 21.34 | weak | 111.5 | 3 | 5 | herbivore | strictly social |
| *Eophona migratoria* | 33.10 | weak | 188.5 | 6 | 4.5 | herbivore | strictly social |
| *Eudynamys scolopaceus* | 31.37 | none | 401.75 | 4 | 1.5 | herbivore | strictly solitary |
| *Pericrocotus roseus* | 31.51 | weak | 186.25 | 4 | 3.5 | carnivore | strictly social |
| *Turdus dissimilis* | 27.46 | weak | 211 | 3 | 3.5 | carnivore | occasionally social |
| *Monticola solitarius* | 28.17 | weak | 207.5 | 4 | 4.5 | carnivore | strictly solitary |
| *Myophonus caeruleus* | 20.72 | weak | 305.5 | 4 | 4 | carnivore | strictly solitary |
| *Schoeniparus dubius* | 8.35 | strong | 136 | 4 | 4 | carnivore | occasionally social |
| *Passer cinnamomeus* | 31.53 | none | 127.75 | 8 | 5 | omnivore | strictly social |
| *Motacilla cinerea* | 35.43 | weak | 179.25 | 4 | 5.1 | carnivore | occasionally social |
| *Erythrogenys gravivox* | 6.75 | strong | 234.25 | 4 | 3 | carnivore | occasionally social |
| *Sinosuthora alphonsiana* | 12.82 | weak | 117.65 | 5 | 4.5 | carnivore | occasionally social |
| *Prinia crinigera* | 10.88 | weak | 147.5 | 7 | 5 | carnivore | occasionally social |
| *Lonchura punctulata* | 21.79 | weak | 112 | 4 | 6 | herbivore | strictly social |
| *Cyanoderma ruficeps* | 9.85 | weak | 107.25 | 4 | 4.5 | carnivore | occasionally social |
| *Spodiopsar sericeus* | 32.62 | none | 215 | 3 | 6.5 | carnivore | strictly social |
| *Terpsiphone incei* | 20.57 | weak | 260.5 | 2 | 3 | carnivore | occasionally social |
| *Pomatorhinus ruficollis* | 10.01 | strong | 169.5 | 5 | 3 | carnivore | occasionally social |
| *Emberiza elegans* | 17.67 | weak | 145 | 5 | 6 | carnivore | strictly social |
| *Dicrurus hottentottus* | 24.47 | strong | 306.75 | 5 | 3.5 | carnivore | strictly solitary |
| *Garrulus glandarius* | 17.51 | weak | 327.5 | 4 | 6.5 | omnivore | strictly social |
| *Eumyias thalassinus* | 20.87 | weak | 148 | 5 | 4 | carnivore | strictly solitary |
| *Muscicapa dauurica* | 29.35 | weak | 120.5 | 4 | 5 | carnivore | occasionally social |
| *Yuhina castaniceps* | 18.95 | weak | 128.5 | 2 | 3.5 | carnivore | strictly social |
| *Emberiza godlewskii* | 21.42 | weak | 157.75 | 5 | 4 | herbivore | occasionally social |
| *Phasianus colchicus* | 22.91 | none | 700 | 4 | 6 | omnivore | strictly social |
| *Emberiza fucata* | 19.49 | weak | 150 | 5 | 5 | omnivore | occasionally social |
| *Psilopogon virens* | 21.67 | weak | 315.75 | 1 | 3.5 | carnivore | occasionally social |
| *Picus canus* | 25.20 | weak | 292.5 | 3 | 9.5 | carnivore | strictly solitary |
| *Pericrocotus cantonensis* | 33.97 | weak | 181.5 | 2 | 4 | carnivore | strictly social |
| *Emberiza cioides* | 23.15 | weak | 158.25 | 4 | 4.5 | carnivore | occasionally social |
| *Culicicapa ceylonensis* | 19.99 | weak | 117 | 5 | 2.5 | carnivore | occasionally social |
| *Phylloscopus trochiloides* | 19.15 | weak | 110.5 | 3 | 5.5 | carnivore | strictly social |
| *Sinosuthora webbiana* | 13.89 | weak | 124.25 | 5 | 4.5 | carnivore | occasionally social |
| *Hierococcyx sparverioides* | 32.72 | none | 384 | 1 | 1.5 | carnivore | strictly solitary |
| *Egretta garzetta* | 33.10 | none | 596.5 | 1 | 4.5 | carnivore | strictly social |
| *Rhyacornis fuliginosa* | 25.41 | weak | 126 | 1 | 4.5 | carnivore | strictly solitary |
| *Ardeola bacchus* | 26.81 | none | 464.25 | 3 | 3 | carnivore | occasionally social |
| *Zapornia akool* | 14.85 | weak | 265 | 1 | 5 | carnivore | strictly social |
| *Tachybaptus ruficollis* | 32.50 | weak | 269 | 1 | 5.5 | carnivore | occasionally social |
| *Alcedo atthis* | 26.86 | weak | 165.5 | 2 | 6 | carnivore | strictly social |
| *Chaimarrornis leucocephalus* | 19.92 | weak | 174.5 | 1 | 3.5 | carnivore | occasionally social |
| *Picumnus innominatus* | 10.66 | strong | 102.5 | 4 | 3.5 | carnivore | strictly solitary |
| *Leiothrix lutea* | 12.75 | weak | 140.25 | 4 | 3.5 | omnivore | strictly social |
| *Ardea cinerea* | 33.22 | none | 888 | 1 | 5 | carnivore | occasionally social |

**Table S5.** Fourth-corner tests of bivariate associations between landscape and habitat characteristics and bird species functional traits. Patch area and PAR were log-transformed. *P*_adj_ represents a *P* value corrected by FDR to account for multiple comparisons.

| Test | Observed | *P*_adj_ |
| --- | --- | --- |
| Patch area / HWI | -0.038 | 0.712 |
| Isolation / HWI | -0.046 | 0.678 |
| PAR / HWI | 0.052 | 0.669 |
| Plant richness / HWI | 0.004 | 0.946 |
| Habitat richness / HWI | -0.005 | 0.946 |
| Patch area / Territoriality | 29.496 | 0.669 |
| Isolation / Territoriality | 14.729 | 0.678 |
| PAR / Territoriality | 27.050 | 0.669 |
| Plant richness / Territoriality | 13.591 | 0.712 |
| Habitat richness / Territoriality | 17.695 | 0.678 |
| Patch area / Body size | -0.066 | 0.601 |
| Isolation / Body size | 0.043 | 0.668 |
| PAR / Body size | 0.057 | 0.601 |
| Plant richness / Body size | -0.055 | 0.601 |
| Habitat richness / Body size | -0.030 | 0.678 |
| Patch area / Habitat specificity | -0.040 | 0.712 |
| Isolation / Habitat specificity | 0.048 | 0.601 |
| PAR / Habitat specificity | 0.034 | 0.712 |
| Plant richness / Habitat specificity | -0.052 | 0.601 |
| Habitat richness / Habitat specificity | -0.043 | 0.601 |
| Patch area / Clutch size | -0.037 | 0.712 |
| Isolation / Clutch size | 0.006 | 0.928 |
| PAR / Clutch size | 0.047 | 0.655 |
| Plant richness / Clutch size | -0.020 | 0.793 |
| Habitat richness / Clutch size | -0.020 | 0.757 |
| Patch area / Trophic level | 5.772 | 0.864 |
| Isolation / Trophic level | 3.472 | 0.876 |
| PAR / Trophic level | 8.986 | 0.793 |
| Plant richness / Trophic level | 5.864 | 0.864 |
| Habitat richness / Trophic level | 1.404 | 0.928 |
| Patch area / Flocking tendency | 29.113 | 0.601 |
| Isolation / Flocking tendency | 4.800 | 0.793 |
| PAR / Flocking tendency | 29.160 | 0.601 |
| Plant richness / Flocking tendency | 14.268 | 0.712 |
| Habitat richness / Flocking tendency | 7.518 | 0.757 |


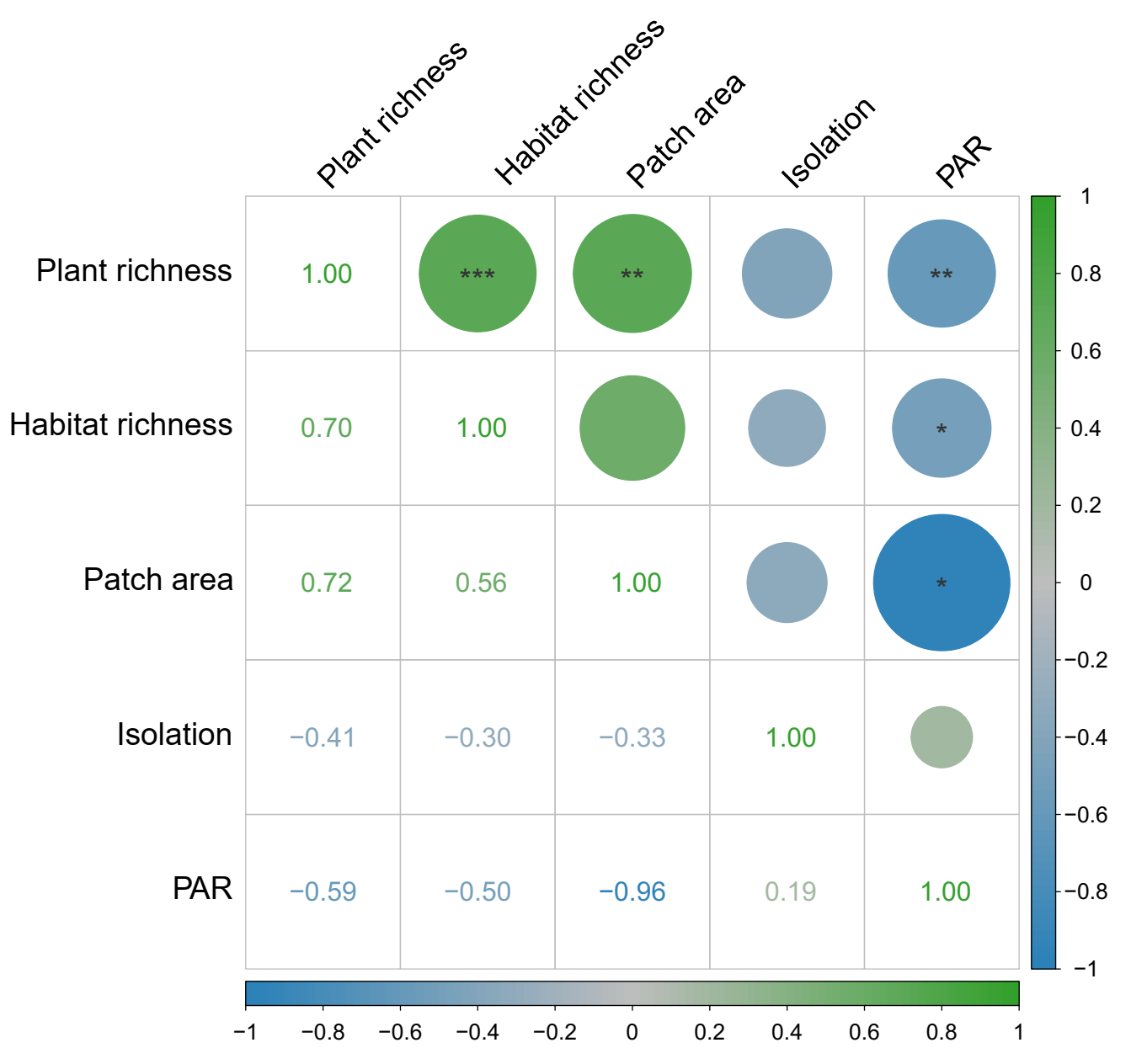


**Fig. S1.** Spearman correlation matrix between landscape and habitat characteristics (including patch area, isolation, PAR, habitat richness, and woody plant richness) of 26 habitat patches in Huaxi University Town, Guizhou, China. Color legend and circles illustrate the correlation values. Significant differences are indicated as * for *P* < 0.05, ** for *P* < 0.01 and *** for *P* < 0.001.


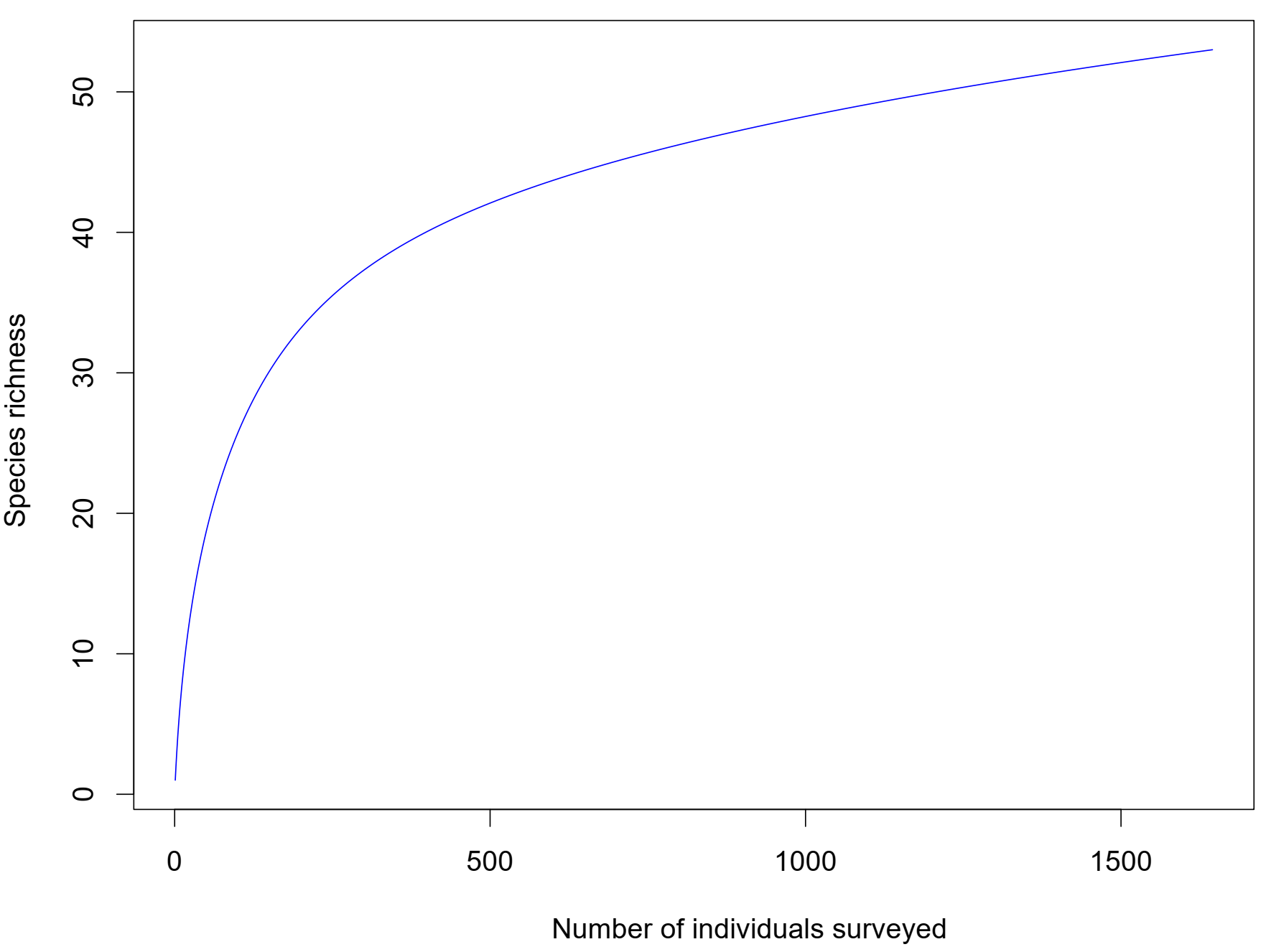


**Fig. S2.** Abundance-based rarefaction curve of bird species for patch No.17 (the largest and proportionally least sampled patch) in Huaxi University Town, Guizhou, China


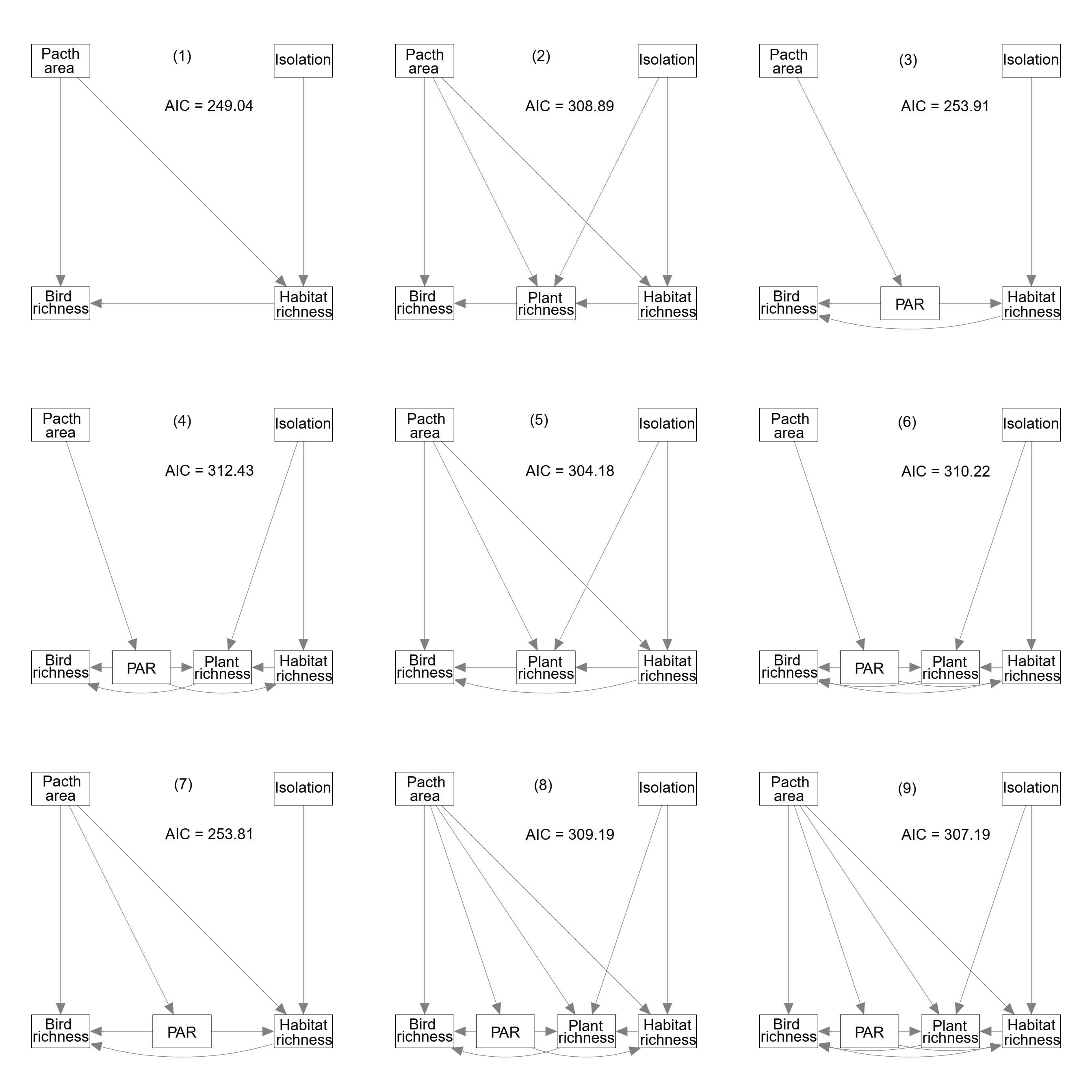


**Fig. S3.** Nine competing models in structural equation modeling. Model 1 included direct effects of patch area and habitat richness on bird richness, with an indirect effect of patch area and isolation via habitat richness. Model 2 included direct effects of patch area and woody plant richness on bird richness with an indirect effect of patch area, isolation and habitat richness via woody plant richness, and an indirect effect of patch area and isolation on woody plant richness via habitat richness. Model 3 included direct effects of PAR and habitat richness on bird richness, with an indirect effect of patch area via PAR and an indirect effect of PAR and isolation via habitat richness. Model 4 included direct effects of PAR and woody plant richness on bird richness with an indirect effect of patch area via PAR and an indirect effect of PAR, isolation and habitat richness via woody plant richness, and an indirect effect of PAR and isolation on woody plant richness via habitat richness. Model 5 incorporated the additive effects of models 1 and 2, while model 6 incorporated the additive effects of models 3 and 4. Alternatively, model 7 incorporated the additive effects of models 1 and 3, while model 8 incorporated the additive effects of models 2 and 4. Finally, Model 9 incorporated the additive effects of models 5, 6, 7, and 8.
